# Antifreeze glycopeptide diastereomers

**DOI:** 10.3762/bjoc.8.190

**Published:** 2012-10-01

**Authors:** Lilly Nagel, Carsten Budke, Axel Dreyer, Thomas Koop, Norbert Sewald

**Affiliations:** 1 Organic and Bioorganic Chemistry, Department of Chemistry, Bielefeld University, Universitätsstraße 25, 33615 Bielefeld, Germany; 2 Physical Chemistry, Department of Chemistry, Bielefeld University, Universitätsstraße 25, 33615 Bielefeld, Germany

**Keywords:** bioorganic chemistry, circular dichroism, glycopeptides, ice recrystallization, microwave chemistry

## Abstract

Antifreeze glycopeptides (AFGPs) are a special class of biological antifreeze agents, which possess the property to inhibit ice growth in the body fluids of arctic and antarctic fish and, thus, enable life under these harsh conditions. AFGPs are composed of 4–55 tripeptide units -Ala-Ala-Thr- glycosylated at the threonine side chains. Despite the structural homology among all the fish species, divergence regarding the composition of the amino acids occurs in peptides from natural sources. Although AFGPs were discovered in the early 1960s, the adsorption mechanism of these macromolecules to the surface of the ice crystals has not yet been fully elucidated. Two AFGP diastereomers containing different amino acid configurations were synthesized to study the influence of amino acid stereochemistry on conformation and antifreeze activity. For this purpose, peptides containing monosaccharide-substituted *allo*-L- and D-threonine building blocks were assembled by solid-phase peptide synthesis (SPPS). The *retro*-*inverso* AFGP analogue contained all amino acids in D-configuration, while the *allo*-L-diastereomer was composed of L-amino acids, like native AFGPs, with replacement of L-threonine by its *allo*-L-diastereomer. Both glycopeptides were analyzed regarding their conformational properties, by circular dichroism (CD), and their ability to inhibit ice recrystallization in microphysical experiments.

## Introduction

Several fish species living in polar and subpolar oceans have developed a strategy to survive in water at an average temperature of −1.8 °C. This non-colligative freezing-point depression is based on the prevention of ice growth in physiological solutions by biological antifreeze agents AFPs (antifreeze proteins) and AFGPs (antifreeze glycoproteins) [1]. This effect is attained by suppressing the nonequilibrium hysteresis freezing point (HFP) with only a minor change in the equilibrium freezing point (EFP) [2–5]. In addition, ice growth is controlled by binding of these macromolecules to the ice surface, thereby inhibiting their growth [1]. AFGPs form a group of biopolymers containing the highly conserved -Ala-Ala-Thr- motif, which is repeated between 4 to 55 times [2,5–8]. Each hydroxy group of the threonine residue is glycosylated with the disaccharide β*-*D-galactosyl-(1→3)-α-*N*-acetyl-D-galactosamine. In spite of the fact that all AFGPs found in arctic and antarctic fish share this structural homology, minor sequence variations occur, such as the replacement of alanine by proline [9–10] or the glycosylated threonine by arginine [11]. Tachibana et al. summarized the essential properties of AFGPs displaying significant antifreeze activity: The disaccharide must be α-glycosidically attached to every threonine; the acetylamino moiety has to be present at the C2 position; the carbohydrates must be *galacto*-configured; and the γ-methyl group of the threonine residue is required [12]. AFGPs are classified as small (AFGP6-8) and large AFGPs (AFGP1-5) depending on their electrophoretic properties. Low-molecular-weight AFGPs are supposed to adopt a threefold left-handed helix, which is highly flexible and similar to polyproline helix type II (PPII) [13]. The larger AFGP1-4 are known to form flexible rods with segmental mobility [14–15].

Recent research has been directed toward the assembly of synthetic AFGPs with tailored amino acid composition and glycosylation pattern. The main target is to investigate the influence of sequential mutations on the adsorption of such synthetic AFGP analogues onto ice surfaces, and to corroborate the molecular mechanism of antifreeze activity. Recently, we published the synthesis of monosaccharide-based AFGP analogues containing glycine, proline and serine instead of alanine residues [16]. These analogues exhibit a decreased antifreeze activity compared to glycopeptides with the native sequence (Ala-Ala-Thr). Similar results were obtained by Peltier et al. with synthetic AFGP analogues comprising both the *N*-acetyl galactosamine as the carbohydrate moiety, and alanine by proline replacements [5]. These peptides exhibit less antifreeze activity than monosaccharide-substituted AFGP analogues without proline residues. Peptoid glycoconjugates with carbohydrate moieties attached by Cu^I^ catalyzed azide-alkyne cycloaddition (CuAAC) were devoid of antifreeze activity [17]. Another type of modification in the AFGP sequence encompasses *C*-linked analogues. For example, galactosyl substituted peptides with different distances between the carbohydrate and the peptide backbone have been reported. The peptides with the shortest distance between the carbohydrate and the amino acid emerged to be potent inhibitors of recrystallization [18]. Furthermore, triazole-connected glycopeptides with different methylene spacers between the carbohydrate moiety and the triazole residue were generated [19]. In this case, the AFGP analogue comprising the smallest distance did not exhibit activity whereas the peptides with one or two methylene spacers showed moderate activity.

## Results and Discussion

In the frame of our ongoing studies on the structure–activity relationship of AFGPs, we became interested in the question of whether an inversion of the configuration at C^β^ of threonine (replacement of L-threonine by *allo*-L-threonine) would lead to AFGP analogues with antifreeze activity. In addition, peptides obtained according to the so-called *retro*-*inverso* concept have attracted considerable interest in the past [20]. These peptides contain all amino acids in D-configuration (*inverso*) and the sequence is inverted (*retro*). Such analogues are supposed to present the amino acid side chains in the same manner as the parent peptides and should, therefore, display analogous activity. Moreover, because of the D-amino acids present, they display, in contrast to their L-configured counterparts, resistance to proteolytic degradation.

Two AFGP mimetics were designed, one comprising exclusively D-configured amino acids and one containing *allo*-L-Thr. For the glycopeptide synthesis the amino acids Fmoc-D-Thr-OH and Fmoc-*allo*-L-Thr-OH were glycosylated with *N*-acetyl galactosamine and incorporated into the peptide by using the stepwise, microwave-enhanced SPPS. Additionally, the corresponding aglycons lacking the carbohydrate moiety were synthesized. The conformation of these synthetic peptides was examined by CD spectroscopic experiments and the antifreeze effect of the glycopeptides was tested in recrystallization assays.

**Scheme 1:**
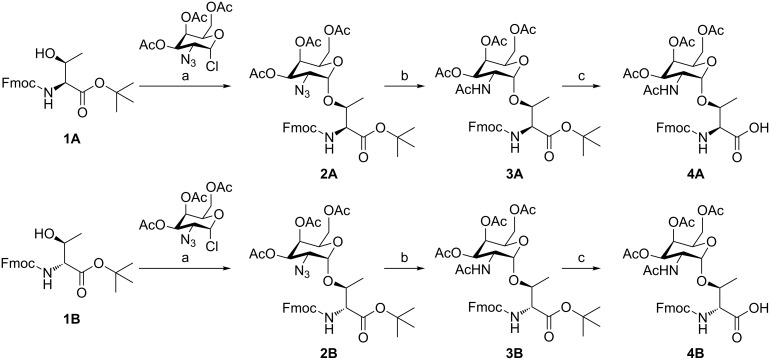
Synthesis of the monosaccharide-substituted threonine building block (**A** = *allo*-L-threonine; **B** = D-threonine). (a) Ag_2_CO_3_, AgClO_4_, CH_2_Cl_2_/toluene (1:1), rt, 40 h, 68% (**A**) and 67% (**B**); (b) AcSH/pyridine (2:1), toluene, 50 °C, 30 min, 56% (**A**) and 42% (**B**); (c) TFA/H_2_O (95:5), 90 min, 96% (**A**) and 90% (**B**).

The glycosylation of threonine in D- and *allo*-L-configuration with *N-*acetyl galactosamine relies on an efficient synthetic procedure by Paulsen et al. [21] adapted by us [16,22–23] (Scheme 1). Azidochlorination of a suitably protected L-galactal provides the peracetylated 2-azido-2-desoxygalactopyranosyl chloride serving as a glycosyl donor in a silver-mediated Koenigs–Knorr glycosylation of the particular Fmoc- and *t*-Bu-protected amino acids, i.e., the *allo*-L-configured (**1A**) and D-configured (**1B**) threonine derivatives. Both glycosylated amino acids (**2**) were obtained in good yields and very good anomeric ratios (**2A**: α/β 9:1; **2B**: α/β 20:1). The azido group was easily reduced and concomitantly acetylated to establish the *N*-acetyl group in the *C*2 position with thioacetic acid in the presence of pyridine [24–25]. The anomers could be easily separated at this stage by column chromatography. In the subsequent step the pure α-product **3A/3B** was subjected to *tert*-butyl ester cleavage with TFA.

Peptide synthesis was accomplished under microwave heating in a semi-automated fashion, as previously published by us [16,23], by employing 2-chlorotrityl resin loaded with Fmoc-L-Ala-OH or Fmoc-D-Ala-OH. After assembly of the glycopeptides the acetate protecting groups of the carbohydrates were cleaved with 5% hydrazine in DMF followed by the cleavage of the peptide from the resin with 2% aqueous TFA. Subsequently, the peptides were precipitated with cold diethyl ether and the solid residues were purified by preparative HPLC. In the case of the aglycons (**6** and **8**) the remaining solids were treated with a high concentration of TFA to cleave the *t*-Bu-protecting groups at the hydroxy group in the side chain of every threonine. These peptides were then purified by HPLC.

**Scheme 2:**
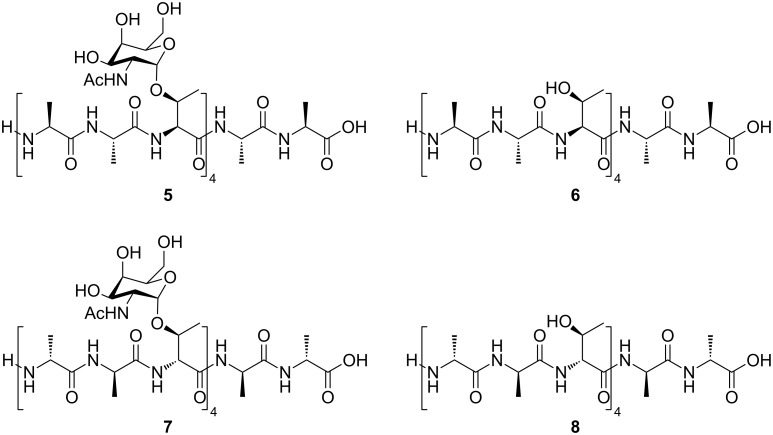
Structures of monosaccharide-substituted AFGP analogues containing *allo*-L-threonine and L-alanine **5**, the corresponding aglycon **6**, the L-glycopeptide comprising D-threonine and D-alanine **7**, and the aglycon **8**.

**Figure 1:**
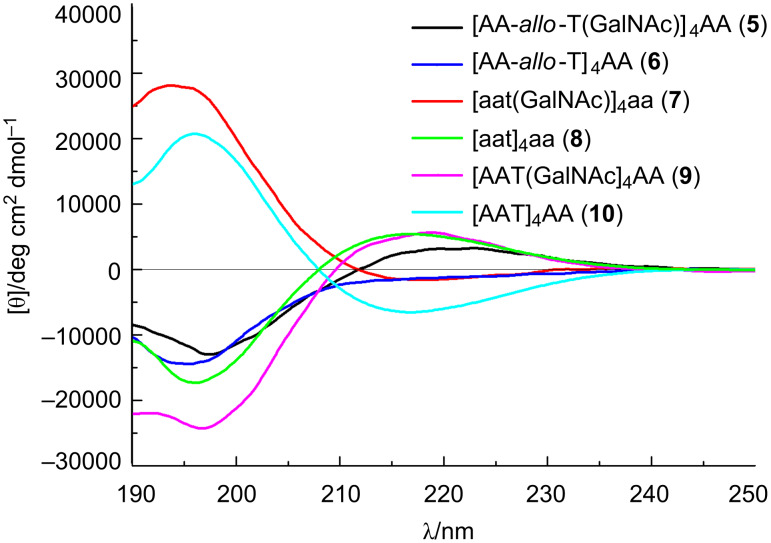
CD spectra of the monosaccharide-substituted AFGP analogues (**5** and **7**) and their corresponding aglycons (**6** and **8**) measured at a concentration of 0.2 mg mL^−1^ in water at 20 °C. For comparison, the spectra of monosaccharide-substituted glycopeptide with naturally occurring sequence **9** and its correlate aglycon **10** are shown [16]. Capital letters symbolize L-amino acids (A = L-Ala, T = L-Thr), lowercase letters D-amino acids (a = D-Ala, t = D-Thr).

**Figure 2:**
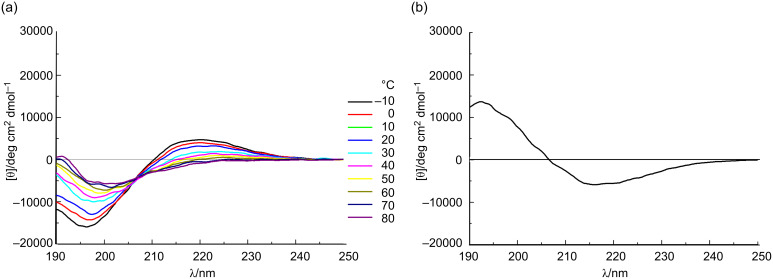
(a) Temperature dependent CD spectra of the glycosylated *allo*-L-Thr containing peptide **5** from −10 to 80 °C in water, revealing an isodichroic point at 207 nm; and (b) the difference spectra between +80 and −10 °C, indicating the contribution of a β-like structure.

**Figure 3:**
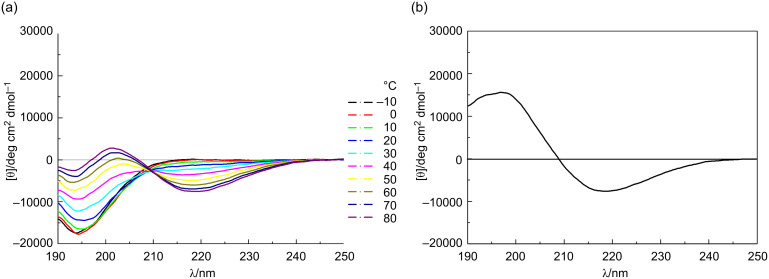
(a) Temperature-dependent CD spectra of the aglycon comprising *allo*-L-Thr **6** from −10 to 80 °C in water, revealing an isodichroic point at 209 nm; and (b) the difference spectra between +80 and −10 °C indicating the contribution of a β-like structure.

The conformations of the *allo*-Thr AFGP analogue **5**, the retro-inverso AFGP analogue **7**, and their corresponding aglycons **6** and **8** (Scheme 2) were analyzed be CD spectroscopy in water at room temperature and additionally at temperatures between −10 and +80 °C. The *allo*-L-threonine containing glycopeptide **5** shows a CD signature typical of a PPII-like conformation with a negative band at λ ≈ 197 nm and a weak positive CD effect at about 220 nm (Figure 1), as already observed for other synthetic AFGP analogues [16,23]. However, the CD intensity is significantly lower compared to the previously published correlate **9** with the native sequence [16]. The (2*S*,3*S*)-configuration of the glycosylated *allo*-L-threonine obviously leads to changes of the conformation compared to glycopeptide **9** containing (2*S*,3*R*)-threonine. The temperature-dependent CD experiments of peptide **5** show an isodichroic point at λ ≈ 207 nm indicating a conformational transition (Figure 2a), presumably between the polyproline II (PPII) helix and the random coil. The difference spectrum generated by subtracting the spectrum at −10 °C from the one recorded at +80 °C indicates a slight increase in β-structures (e.g., β-turns, Figure 2b) [26–27]. Glycopeptide **5** presumably undergoes a local transition from a conformational mixture of PPII and random coil at low temperature to a combination of β*-*structures and random coil at higher temperature [28–30]. In contrast, the corresponding aglycon **6** shows a negative CD effect at λ ≈ 195 nm comparable to **5** but no positive band (Figure 1). The temperature-dependent CD experiments reveal an isodichroic point at λ ≈ 209 nm indicating a conformational equilibrium (Figure 3a). The difference spectrum reveals a typical β-sheet- or β-strand-like structure (Figure 3b). The propensity to adopt a PPII helical structure is reduced in the aglycon **6** compared to the glycopeptide **5**. The high β-sheet- and β-strand-like character is already visible in the CD curve recorded at 50 °C (Figure 3a). Interestingly, unlike the aglycon **10**, which contains L-threonine, the aglycon **6** containing *allo*-L-threonine does not as readily adopt a β-sheet-like structure at room temperature (Figure 1) [16]. The difference in configuration at C^β^ of the threonine residue seems to reduce the propensity for β-strand formation. In the φ/ψ-energy map the conformers PPII and β-strand are very close, and the energy barrier is quite low, facilitating the transition between the conformers [31–32].

**Figure 4:**
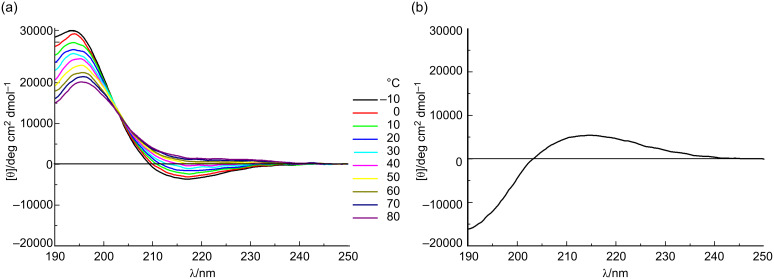
(a) Temperature-dependent CD spectra of the glycosylated D-Thr and D-Ala containing peptide **7** from −10 to 80 °C in water, revealing an isodichroic point at 203 nm; and (b) the difference spectra between +80 and −10 °C.

For the *retro*-*inverso* peptides **7** and **8**, containing exclusively D-configured amino acids, contrasting results were observed. The CD spectrum of AFGP analogue **7** is nearly a mirror image to the CD spectrum obtained for glycopeptide **9** with almost equivalent ellipticities but, as expected for a D-configured peptide, opposite signs (Figure 1). Small deviations in the CD spectrum of **7** can be attributed to the influence of the carbohydrate moieties, which are D-configured in both glycopeptides. The absolute intensity of the band at 194 nm of the *retro*-*inverso* peptide **7** is slightly increased compared to that of **9**, while it is less intense for the band of **7** at 217 nm. This may be attributed to decreased PPII character of **7**. Temperature-dependent CD spectra reveal an isodichroic point at λ ≈ 203 nm indicating a conformational transition (Figure 4a). The difference spectrum generated by subtracting the CD spectra of −10 from +80 °C exhibits a curve indicating a transition from PPII/random coil structures at lower temperatures to an increasing proportion of β-turn structures (Figure 4b). Aglycon **8**, the enantiomer of the all-L-peptide **10**, exhibits the mirror image CD spectrum (Figure 1) [33]. Furthermore, the peptide conformation is temperature-independent as judged by the absence of significant changes of the CD spectrum with increasing temperature. The aglycon **8** adopts only β-sheet structure as shown for the aglycon **10** containing exclusively L-amino acids [16].

**Figure 5:**
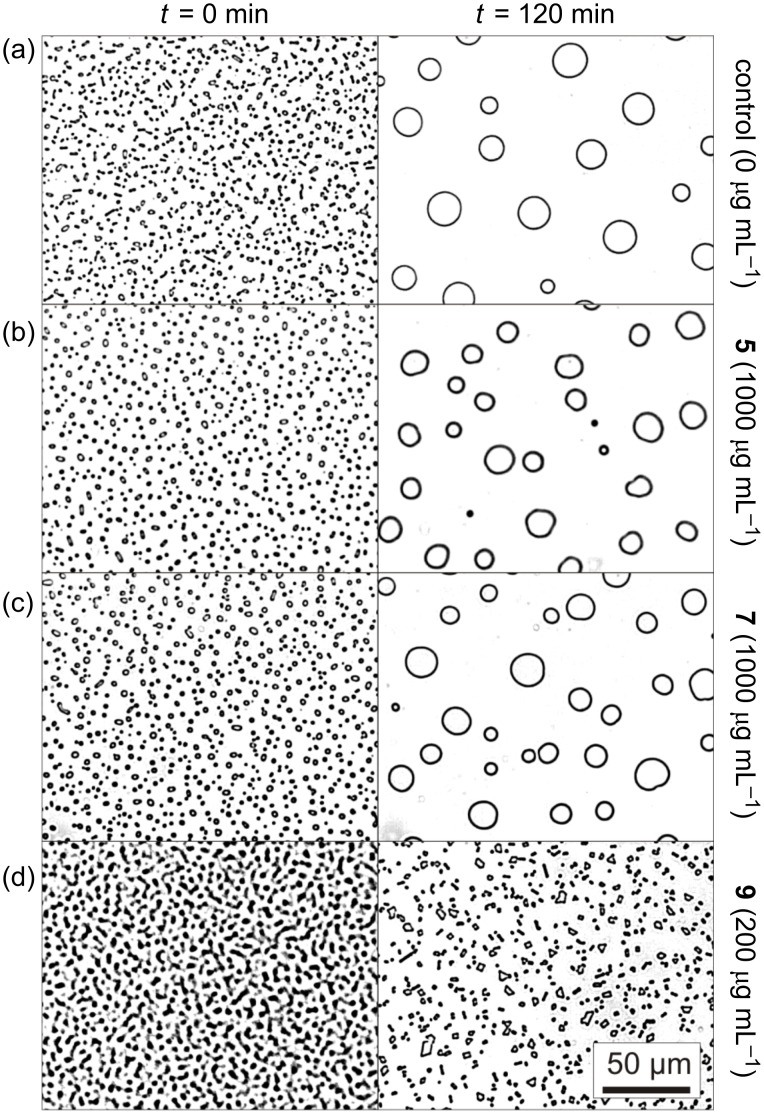
Optical microphotographs taken after 0 and 120 min during the recrystallization process of polycrystalline ice samples at −8 °C formed in aqueous 45 wt % sucrose solutions. (a) Negative control solution without peptides. (b) Peptide **5** (*c* 1000 µg mL^−1^). (c) Peptide **7** (*c* 1000 µg mL^−1^). (d) Positive control solution containing 200 µg mL^−1^ of the ice recrystallization inhibiting peptide **9** ([AAT(GalNAc)]_4_AA). The pictures were contrast-enhanced for better visibility.

The *allo*-L-threonine analogue **5** and the *retro*-*inverso* analogue **7** were tested in this study in an ice-recrystallization-inhibition assay [34–37]. In the absence of any active additives a polycrystalline ice sample undergoes Ostwald ripening at constant temperatures driven by the reduction of the total ice/solution interface energy. During this recrystallization process the amount of ice stays constant while the number of crystals decreases, and hence, the average size increases (Figure 5a). The rate of this process is controlled predominantly by the diffusion of water molecules between the adjacent ice crystals. In the presence of ice-binding antifreeze agents, however, the limiting factor becomes the liquid-to-ice transfer. In the case of sufficiently large concentrations of antifreeze agents the recrystallization is retarded or even entirely inhibited. The monoglycosylated peptide **9** ([AAT(GalNAc)]_4_AA), comprising L-amino acids, reduced the recrystallization rate already at concentrations of about *c*_i_ ≈ 20 µg mL^−1^ (0.01 mM) [37]. Figure 5d shows the result of an experiment at *c* ≈ 200 µg mL^−1^ of peptide **9**, at which the ripening is totally inhibited after a few minutes. In contrast, the peptides **5** and **7** do not inhibit ice recrystallization significantly in the investigated concentration range from 100 up to 1000 µg mL^−1^ (0.5 mM, Figure 5b,c). After 120 min the crystal number had decreased and the average size had increased by Ostwald ripening similar to the control solution without any peptides.

The propensity of an AFGP analogue to adopt PPII-helical structure is obviously not the only precondition to qualify it as an antifreeze agent [38]. The GalNAc-*allo*-Thr containing peptide **5** forms a PPII helix, albeit with decreased propensity. However, **5** is devoid of antifreeze activity, as it does not inhibit ice recrystallization. The *retro*-*inverso* AFGP analogue **7** adopts a PPII helix with opposite helicity. The D-configured peptide backbone might, therefore, qualify for interaction with the achiral ice surface. However, the combination of a D-configured PPII helical peptide with the native D-GalNAc residues abolishes antifreeze activity. Although the peptide backbone of **7** is the mirror image of the native parent compound **9**, which adopts a right-handed helix, no activity was obtained for the *retro*-*inverso* analogue **7**. The different behavior can be explained only by a change in the conformational presentation of the GalNAc residue and altered interaction pattern (e.g., by hydrogen bonds) between peptide and carbohydrate. According to the literature, model studies have provided indications that a carbohydrate such as *N*-acetyl-D-galactosamine influences the conformation by interactions such as hydrogen bonding and the presence of water pockets/bridges between the monosaccharide and the peptide backbone [39–40]. Hence, both configurational and conformational elements of the glycopeptides are essential for antifreeze activity. The (2*S*,3*S*)-configured *allo*-L-threonine may direct the carbohydrate moiety into an unfavorable position for adsorption to the ice surface; a similar situation applies to the *retro*-*inverso* peptide **7**. Moreover, these conformational changes may possibly also alter the water hydration shell around the peptide, which has been suggested to be essential for the antifreeze activity of AFGPs and AFGP analogues [41–42].

## Conclusion

A GalNAc-substituted AFGP analogue [AA-*allo*-T(GalNAc)]_4_AA (**5**) and a *retro*-*inverso* AFGP analogue [aat(GalNAc)]_4_aa (**7**) with GalNAc-D-Thr and D-Ala building blocks were synthesized. The conformations of these glycopeptides and their corresponding aglycons were investigated by CD spectroscopy. According to these data, both peptides adopt polyproline II helical conformations, albeit with reduced propensity (**5**) or opposite helicity (**7**).

The structure of the glycopeptides was correlated with their ability to inhibit ice growth in microphysical recrystallization assays. Both AFGP analogues **5** and **7** did not show antifreeze activity. Hence, the altered configuration at Thr C^β^ of **5** or at Thr C^α^/C^β^ of **7** may position the carbohydrate residue differently with implications on the formation of hydrogen bonds and the presence of water pockets/bridges between the monosaccharide and the peptide backbone as well as on the hydration shell around the peptide.

## Experimental

All chemicals were acquired from Sigma Aldrich (Hamburg, Germany), Acros (Geel, Belgium), Alfa Aesar (Ward Hill, USA) and VWR (Darmstadt, Germany) and were employed without additional purification. Moisture- and air-sensitive reaction steps were conducted in flame-dried glassware under an argon atmosphere. Dichloromethane and toluene were freshly distilled from CaH_2_ and Na, respectively. Dimethylformamide was distilled from ninhydrin. 2-Chlorotrityl resin ChemMatrix resin (0.6 mmol/g), coupling reagents and all amino acids were purchased from Iris Biotech (Marktredwitz, Germany), Orpegen (Heidelberg, Germany) and Molekula (Gillingham, Dorset, UK). Peptide synthesis was executed on a Liberty Automated Microwave Peptide Synthesizer (CEM, Kamp-Lintfort, Germany). For preparative RP-HPLC a Thermo Separation Products system equipped with a UV-1000 detector, a P-4000 pump, a Vydac high-performance guard column (C18) and a Phenomenex Jupiter 10 µ Proteo 90 Å column (C12; 250 × 21.20 mm) or a Hitachi Merck LaChrom system equipped with a UV–vis L-7420 detector and a L-7150 pump equipped with a Vydac high-performance guard column (C18) and a Phenomenex Jupiter 10 µ 300 Å column (C18; 250 × 21.20 mm) was used. A flow rate of 7.5 mL min^−1^ using eluent A: H_2_O/CH_3_CN/TFA (98:1.95:0.05) and eluent B: CH_3_CN/H_2_O/TFA (95:4.9:0.1) was employed. The gradient was executed within 30 min from 100 to 90% eluent B ending after 60 min at 50% eluent B. MALDI-TOF mass spectra were measured on a Voyager DE Instrument (PE Biosystems, Weiterstadt, Germany) mounted with a 1.2 m flight tube. 2,5-Dihydroxybenzoic acid was used as the matrix. Depending on the mass range the ions were accelerated at 15 to 25 kV with the option of detecting positive or negative ions. The instrument default calibration was used for calibrating the mass axis. ESI experiments were performed on a Fourier Transform Ion Cyclotron Resonance (FT-ICR) mass spectrometer APEX III (Bruker Daltonik, Bremen, Germany) equipped with a 7.0 T, 160 mm bore superconducting magnet (Bruker Analytik GmbH – Magnetics, Karlsruhe, Germany), infinity cell, and interfaced to an external (nano)ESI or MALDI ion source. Scan accumulation and Fourier transformation were done with XMASS NT (7.08) on a PC workstation; for further data processing DataAnalysis^TM^ 3.4 was used. Optical rotation was measured on a DIP-360 digital polarimeter (Jasco, Groß-Umstadt, Germany) at 20–23 °C. NMR spectra were recorded on a DRX 500 and an Avance 600 spectrometer (Bruker Biospin, Rheinstetten, Germany). CD spectra were obtained on a J-810 spectrometer equipped with a CDF-4265 Peltier unit for temperature control (Jasco, Groß-Umstadt, Germany). The spectra were recorded in the range of 190–250 nm at a scanning rate of 50 nm min^−1^ with three accumulations, a data pitch of 0.2 nm, a spectral band width of 1 nm and a response time of 1 s. The peptide solutions had a concentration of 0.2 mg mL^−1^ and were measured in a 0.1 cm quartz cell. Molar ellipticity per amino acid residue [θ]_mr_ was calculated as follows: [θ]_mr_ = θ/(10*•N•c•l*). θ represents the ellipticity in millidegrees, *N* the number of amino acid residues, *c* the molar concentration in moles per liter, and *l* the cell path length in centimeters. Temperature dependent spectra were measured in a range of −10 to 80 °C by increasing the temperature in intervals of 5 °C. The temperature slope was 1 °C min^−1^ with an equilibration time of 5 min. The CD spectra were smoothed by a Savitzky–Golay method.

### General synthetic procedure for the glycosylation step (GP1)

The glycosyl acceptors Fmoc-L-*allo*-Thr-O*t-*Bu (**1A**) and Fmoc-D-Thr-O*t*-Bu (**1B**, 1.0 equiv), respectively, and Ag_2_CO_3_ (1.5 equiv) were suspended with freshly activated, powdered 4 Å molecular sieves in abs toluene (30–40 mL) and methylene chloride (30–40 mL) at –20 °C and stirred for 30 min. Then, the silver salt promoter AgClO_4_ (0.25 equiv) was added at room temperature and stirring was continued for 30 min. Subsequently, the 2-azido-2-deoxy-3,4,6-tri-*O*-acetyl-α-D-galactopyranosyl chloride (1.5 equiv) was added dissolved in abs toluene (30–40 mL) and methylene chloride (30–40 mL), and the solution was stirred in the dark under argon atmosphere at room temperature overnight. The mixture was diluted with methylene chloride (140–200 mL), filtered through Celite and washed with water (70–100 mL) and aqueous saturated NaHCO_3_ solution (50–70 mL). The organic layer was dried over Na_2_SO_4_, and the solvent was removed in vacuo. The crude product was purified by column chromatography (PE/EtOAc; 2:1) yielding a white solid.

***N*-[(9*H*-Fluoren-9-yl)methoxycarbonyl]-3-*O*-(2-azido-2-deoxy-3,4,6-tri-*O*-acetyl-α-D-galactopyranosyl)-L-*allo*-threonine *tert*-butyl ester (2A):** Yield from **1A** (3.00 g, 8.6 mmol) using GP1: 4.17 g (5.9 mmol, 68%) in α/β ratio 9:1; [α]_D_^20^ +50.9 (*c* 0.45, CHCl_3_); ^1^H NMR (500 MHz, CDCl_3_) δ 1.42 (d, *J* = 6.6 Hz, 3H, Hγ-Thr), 1.54 (s, 9H, CH_3_-*t*-Bu), 2.02 (s, 3H, CH_3_CO), 2.07 (s, 3H, CH_3_CO), 2.18 (s, 3H, CH_3_CO), 3.61 (dd, *J* = 11.3, 3.4 Hz, 1H, H2-Gal), 4.12–4.15 (m, 3H, H6-Gal, Hβ-Thr), 4.25 (m, 1H, H5-Gal), 4.41–4.45 (m, 3H, Hα-Thr, CH_2_-Fmoc), 5.01 (d, *J* = 3.4 Hz, 1H, H1-Gal), 5.35 (dd, *J* = 3.1, 11.3 Hz, 1H, H3-Gal), 5.48 (m, *J* = 3.0 Hz, 1H, H4-Gal), 5.72 (d, *J* = 6.3 Hz, 1H, CH-Fmoc), 6.06 (d, *J* = 8.1 Hz, 1H, NH-Thr), 7.31–7.45 (m, 4H, aryl-H), 7.61–7.67 (m, 2H, aryl-H), 7.76–7.81 (m, 2H, aryl-H); ^13^C NMR (125.8 MHz, CDCl_3_) δ 14.2 (Hγ-Thr), 20.7 (CH_3_CO), 21.1 (2× CH_3_CO), 28.1 (CH_3_-*t*-Bu), 47.2 (CH-Fmoc), 59.1 (C2-Gal), 59.8 (Cα-Thr), 61.9 (C5-Gal), 67.3 (C4-Gal), 67.8 (CH_2_-Fmoc, C6-Gal), 69.8 (C3-Gal), 78.4 (Cβ-Thr), 83.3 (C-*t*-Bu), 98.4 (C1-Gal), 120.0 (2×), 125.1 (2×), 127.1 (2×), 127.7 (2×), 141.3 (2×), 143.8 (2×) (aryl), 156.7 (CO-Fmoc), 168.1, 169.6, 170.0, 170.5 (CO); MS (ESI) *m*/*z*: [M + Na]^+^ 733.3.

***N*-[(9*H*-Fluoren-9-yl)methoxycarbonyl]-3-*O*-(2-azido-2-deoxy-3,4,6-tri-*O*-acetyl-α-D-galactopyranosyl)-D-threonine *tert*-butyl ester (2B):** Yield from **1B** (5.00 g, 12.6 mmol) using GP1: 5.99 g (8.4 mmol, 67%) in α/β ratio 20:1; [α]_D_^20^ +73.7 (*c* 0.5, CHCl_3_); ^1^H NMR (500 MHz, CDCl_3_): δ 1.29 (d, *J* = 6.2 Hz, 3H, H*γ*-Thr), 1.51 (s, 9H, CH_3_-*t*-Bu), 2.03 (s, 3H, CH_3_CO), 2.08 (s, 3H, CH_3_CO), 2.14 (s, 3H, CH_3_CO), 3.61 (dd, *J* = 11.2, 3.5 Hz, 1H, H2-Gal), 4.03 (m, 1H, H5-Gal), 4.09–4.15 (m, 2H, H6-Gal), 4.21–4.27 (m, 2H, CH_2_-Fmoc, CH-Fmoc), 4.39–4.45 (m, 3H, Hα-Thr, Hβ-Thr, CH_2_-Fmoc), 5.17 (d, *J* = 3.6 Hz, 1H, H1-Gal), 5.31 (m, 1H, H3-Gal), 5.42 (m, 1H, H4-Gal), 5.54 (d, *J* = 9.5 Hz, 1H, NH-Thr), 7.29–7.42 (m, 4H, aryl-H), 7.60–7.63 (m, 2H, aryl-H), 7.75–7.78 (m, 2H, aryl-H); ^13^C NMR (125.8 MHz, CDCl_3_) δ 15.0 (Cγ-Thr), 20.8 (2× CH_3_CO), 21.2 (CH_3_CO), 28.1 (CH_3_-*t*-Bu), 47.3 (CH-Fmoc), 57.4 (C2-Gal), 59.2 (Cα-Thr), 60.5 (C5-Gal), 61.7 (C6-Gal), 67.4 (CH_2_-Fmoc), 67.5 (C3-Gal), 67.7 (C4-Gal), 72.6 (Cβ-Thr), 83.3 (C-*t*-Bu), 95.0 (C1-Gal), 120.0 (2×), 125.3 (2×), 127.2 (2×), 127.9 (2×), 141.5 (2×), 143.9, 144.0 (aryl), 156.7 (CO-Fmoc), 169.3, 169.9, 170.2, 170.5 (CO); MS (ESI) *m*/*z*: [M + Na]^+^ 733.2.

### General synthetic procedure for the simultaneous reduction and *N*-acetylation step (GP2)

Fmoc-L-*allo*-Thr(α/β-Ac_3_GalN_3_)-O*t*-Bu (**2A**) or Fmoc-D-Thr(α/β-Ac_3_GalN_3_)-O*t*-Bu (**2B**) was dispensed in a mixture of thioacetic acid and pyridine (2:1) and stirred at 50 °C for 30 min. The solvents were distilled off azeotropically three times with toluene. The residue was purified by flash chromatography (PE/EtOAc; 1:1) to give diastereochemically pure α-configured product.

***N*-[(9*H*-Fluoren-9-yl)methoxycarbonyl]-3-*O*-(2-acetamido-2-deoxy-3,4,6-tri-*O*-acetyl-α-D-galactopyranosyl)-L-*allo*-threonine *tert*-butyl ester (3A):** Yield from **2A** (4.17 g, 5.9 mmol) using GP2: 2.40 g (3.3 mol, 56%); [α]_D_^20^ +72.2 (c 0.69, CHCl_3_); ^1^H NMR (500 MHz, CDCl_3_) δ 1.30 (d, *J* = 7.2 Hz, 3H, Hγ-Thr), 1.54 (s, 9H, CH_3_-*t*-Bu), 1.96 (s, 3H, CH_3_CO), 2.02 (s, 3H, CH_3_CO), 2.07 (s, 3H, CH_3_CO_NAc_), 2.18 (s, 3H, CH_3_CO), 4.08 (m, *J* = 6.8 Hz, 1H, Hβ-Thr), 4.09–4.12 (m, 2H, H6-Gal), 4.25 (t, *J* = 6.8 Hz, 1H, CH-Fmoc), 4.40–4.46 (m, 4H, Hα-Thr, CH_2_-Fmoc, H5-Gal), 4.57 (m, 1H, H2-Gal), 4.98 (d, *J* = 3.1 Hz, 1H, H1-Gal), 5.13 (m, 1H, H3-Gal), 5.41 (m, 1H, H4-Gal), 5.61 (d, *J* = 9.1 Hz, 1H, NH-Gal), 5.93 (d, *J* = 7.4 Hz, 1H, NH-Thr), 7.32–7.45 (m, 4H, aryl-H), 7.62–7.66 (m, 2H, aryl-H), 7.77–7.81 (m, 2H, aryl-H); ^13^C NMR (125.8 MHz, CDCl_3_) δ 14.2 (Cγ-Thr), 20.7 (CH_3_CO), 20.8 (2× CH_3_CO), 23.3 (CH_3_CO), 28.1 (CH_3_-*t*-Bu), 47.1 (CH-Fmoc), 48.0 (C2-Gal), 58.8 (Cα-Thr), 62.0 (C6-Gal), 67.1 (CH_2_-Fmoc), 67.4 (C4-Gal, C5-Gal), 68.3 (C3-Gal), 76.1 (Cβ-Thr), 83.1 (C-*t*-Bu), 96.6 (C1-Gal), 120.0 (2×), 125.1 (2×), 127.1 (2×), 127.7 (2×), 141.3 (2×), 143.8 (2× aryl), 155.9 (CO-Fmoc), 168.3, 169.9, 170.5, 170.0, 170.2 (CO); MS (ESI) *m*/*z*: [M + Na]^+^ 749.1.

***N*-[(9*H*-Fluoren-9-yl)methoxycarbonyl]-3-*O*-(2-acetamido-2-deoxy-3,4,6-tri-*O*-acetyl-α-D-galactopyranosyl)-D-threonine *tert*-butyl ester (3B):** Yield from **2B** (5.99 g, 8.4 mmol) using GP2: 2.57 g (3.5 mol, 42%); [α]_D_^20^ +58.2 (*c* 0.5, CHCl_3_); ^1^H NMR (500 MHz, CDCl_3_) δ 1.18 (d, *J* = 7.2 Hz, 3H, Hγ-Thr), 1.48 (s, 9H, CH_3_-*t*-Bu), 1.95 (s, 3H, CH_3_CO), 1.99 (s, 3H, CH_3_CO), 2.01 (s, 3H, CH_3_CO), 2.16 (s, 3H, CH_3_CO), 4.03 (m, *J* = 9.8, 6.6 Hz, 1H, H5-Gal), 4.09–4.15 (m, 2H, H6-Gal), 4.18–4.26 (m, 2H, CH-Fmoc, Hβ-Thr), 4.33 (dd, *J* = 9.3, 3.5 Hz, 1H, Hα-Thr), 4.48–4.60 (m, 3H, CH_2_-Fmoc, H2-Gal), 5.01 (d, *J* = 3.6 Hz, 1H, H1-Gal), 5.06 (dd, *J* = 11.4, 3.2 Hz, 1H, H3-Gal), 5.31 (m, 1H, H4-Gal), 5.57 (d, *J* = 9.2 Hz, 1H, NH-Thr), 5.90 (d, *J* = 7.4 Hz, 1H, NH-Gal), 7.29–7.44 (m, 4H, aryl-H), 7.58–7.65 (m, 2H, aryl-H), 7.75–7.79 (m, 2H, aryl-H); ^13^C NMR (125.8 MHz, CDCl_3_) δ 15.5 (Cγ-Thr), 20.7 (CH_3_CO), 20.9 (2× CH_3_CO), 23.3 (CH_3_CO), 28.1 (CH_3_-*t*-Bu), 47.4 (CH-Fmoc), 47.8 (C2-Gal), 59.2 (Cα-Thr), 62.0 (C5-Gal), 66.9 (C6-Gal), 67.3 (CH_2_-Fmoc), 67.4 (C4-Gal), 68.3 (C3-Gal), 72.5 (Cβ-Thr), 83.3 (C-*t*-Bu), 94.5 (C1-Gal), 120.2 (2×), 125.0, 125.1, 127.3 (2×), 127.9 (2×), 141.5 (2×), 143.9, 144.0 (aryl), 156.6 (CO-Fmoc), 169.5, 170.3, 170.5, 171.1, 171.3 (CO); MS (ESI) *m*/*z*: [M + Na]^+^ 749.3.

### General synthetic procedure for the *tert*-butyl deprotection step (GP3)

Fmoc-L-*allo*-Thr(GalNAc)-O*t*-Bu (**3A**) or Fmoc-L-*allo*-Thr(GalNAc)-O*t*-Bu (**3B**) was dissolved in a mixture of TFA and water (95:5) and stirred for 90 min. The volatiles were evaporated and the remaining residue was suspended in a mixture of acetonitrile and water and lyophilized three times in this solvent mixture. The crude product **4** was used without further purification.

***N*-[(9*H*-Fluoren-9-yl)methoxycarbonyl]-3-*O*-(2-acetamido-2-deoxy-3,4,6-tri-*O*-acetyl-α-D-galactopyranosyl)-L-*allo*-threonine (4A):** Yield from **3A** (2.40 g, 3.3 mmol) using GP3: 2.13 g (3.2 mmol, 96%); [α]_D_^20^ +70.4 (*c* 0.6, CHCl_3_); ^1^H NMR (500 MHz, DMSO-*d*_6_) δ 1.10 (d, *J* = 6.2 Hz, 3H, Hγ-Thr), 1.82 (s, 3H, CH_3_CO), 1.89 (s, 3H, CH_3_CO), 1.91 (s, 3H, CH_3_CO), 2.08 (s, 3H, CH_3_CO), 3.89–3.92 (m, 2H, H6-Gal), 4.01–4.09 (m, 3H, Hβ-Thr, CH_2_-Fmoc), 4.18 (m, 1H, H2-Gal), 4.22–4.30 (m, 3H, Hα-Thr, H5-Gal, CH-Fmoc), 4.95 (dd, *J* = 11.9, 3.1 Hz, 1H, H3-Gal), 5.03 (d, *J* = 3.5 Hz, 1H, H1-Gal), 5.27 (m, 1H, H4-Gal), 7.31–7.44 (m, 4H, aryl-H), 7.72–7.76 (m, 2H, aryl-H), 7.77 (d, *J* = 8.9 Hz, 1H, NH-Thr), 7.88–7.91 (m, 2H, aryl-H), 7.96 (d, *J* = 7.8 Hz, 1H, NH-Gal), 12.91 (s, br, 1H, COOH); ^13^C NMR (125.8 MHz, DMSO-*d*_6_) δ 15.5 (Cγ-Thr), 20.3 (CH_3_CO), 20.4 (CH_3_CO), 20.5 (CH_3_CO), 22.3 (CH_3_CO), 46.6 (C2-Gal), 47.2 (CH-Fmoc), 58.6 (CH_2_-Fmoc), 61.2 (C6-Gal), 65.9 (Cα-Thr), 66.4 (C5-Gal), 66.9 (C4-Gal), 67.3 (C3-Gal), 73.6 (Cβ-Thr), 95.7 (C1-Gal), 120.1 (2×), 125.3 (2×), 127.0 (2×), 127.6 (2×), 140.7 (2×), 143.7 (2× aryl), 156.2 (CO-Fmoc), 169.7 (3×), 169.9, 171.7 (CO); MS (ESI) *m*/*z*: [M + Na]^+^ 693.1.

***N*-[(9*H*-Fluoren-9-yl)methoxycarbonyl]-3-*O*-(2-acetamido-2-deoxy-3,4,6-tri-*O*-acetyl-α-D-galactopyranosyl)-D-threonine (4B):** Yield from **3B** (2.57 g, 3.5 mmol) using GP3: 2.14 g (3.2 mmol, 90%). [α]_D_^20^ +59.7 (*c* 0.5, CHCl_3_); ^1^H NMR (500 MHz, DMSO-*d*_6_) δ 1.02 (d, *J* = 6.2 Hz, 3H, H*γ*-Thr), 1.85 (s, 3H, CH_3_CO), 1.92 (s, 3H, CH_3_CO), 1.97 (s, 3H, CH_3_CO), 2.09 (s, 3H, CH_3_CO), 3.88 (dd, *J* = 9.9, 5.1 Hz, 1H, H6a-Gal), 4.07–4.13 (m, 2H, H5-Gal, H6b-Gal), 4.21–4.30 (m, 3H, Hβ-Thr, CH_2_-Fmoc), 4.30–4.45 (m, 3H, H2-Gal, Hα-Thr, CH-Fmoc), 4.88 (d, *J* = 3.7 Hz, 1H, H1-Gal), 4.94 (dd, *J* = 11.6, 3.3 Hz, 1H, H3-Gal), 5.25 (m, *J* = 3.3 Hz, 1H, H4-Gal), 7.26–7.44 (m, 4H, aryl-H), 7.62 (d, *J* = 8.6 Hz, 1H, NH-Gal), 7.70–7.77 (m, 2H, aryl-H), 7.87–7.91 (m, 2H, aryl-H), 8.01 (d, *J* = 9.3 Hz, 1H, NH-Thr), 13.03 (s, br, 1H, COOH); ^13^C NMR (125.8 MHz, DMSO-*d*_6_) δ 14.4 (Cγ-Thr), 20.4 (CH_3_CO), 20.5 (2× CH_3_CO), 22.5 (CH_3_CO), 45.4 (C2-Gal), 47.3 (CH-Fmoc), 58.3 (Cα-Thr), 61.1 (C6-Gal), 65.7 (CH_2_-Fmoc), 66.2 (C5-Gal), 66.8 (C4-Gal), 67.9 (C3-Gal), 70.6 (Cβ-Thr), 92.9 (C1-Gal), 120.1 (2×), 125.1, 125.3, 127.0 (2×), 127.7 (2×), 140.8 (2×), 143.7, 144.0 (aryl), 156.8 (CO-Fmoc), 169.4, 169.8, 170.0 (2×), 172.0 (CO); MS (ESI) *m*/*z*: [M + Na]^+^ 693.2, [M + K]^+^ 715.1.

### Synthesis of AFGP analogues and their aglycons

Synthesis of the peptides was conducted in a microwave reactor in an automatic fashion for the aglycons (**6** and **8**) and semi-automatically for the AFGP analogues (**5** and **7**) on a 0.10 mmol scale. The 2-chlorotrityl resin was loaded manually either with Fmoc-L-Ala-OH (resin loading 0.59 mmol g^−1^) for the *allo*-L-Thr containing peptides or with Fmoc-D-Ala-OH (resin loading 0.38 mmol g^−1^) for the *retro*-*inverso* peptides. All cycles for the synthesis of the glycosylated peptides and aglycons follow a published procedure [16,23]. The automated synthesis of the aglycons was performed under microwave irradiation at a maximum of 78 °C at 35 W, whereas for the glycopeptides lower temperatures limited to 40 °C at 20 W were employed. The resin was treated twice with piperidine in DMF (20%, v/v, 7 mL) for 7 min to cleave Fmoc. Coupling of Fmoc-L/D-Ala-OH and Fmoc-L/D-Thr(*t*-Bu)-OH, (0.50 mmol, 5.0 equiv) was conducted in 10 min by using a solution of the particular amino acid in DMF (2.5 mL), TBTU (0.50 mmol, 5.0 equiv) in DMF (1 mL) and DIPEA (1.0 mmol, 10 equiv) in NMP (0.5 mL). Fmoc-*allo*-L/D-Thr(Ac_3_GalNAc)-OH **4A**/**B** (0.25 mmol, 2.5 equiv) were preactivated with HATU (0.28 mmol 2.8 equiv) in DMF (1 mL) and DIPEA (0.28 mmol, 2.75 equiv) for 2 min and added manually to the reaction vessel of the peptide synthesizer. Subsequently, HOAt (0.25 mmol, 2.25 equiv) dissolved in DMF (1 mL) and DIPEA (0.28 mmol, 2.75 equiv) were transferred manually. Unreacted amino groups were capped by an acetylation reaction after coupling of the glycosylated building block using acetic anhydride (0.5 mol) in the presence of DIPEA (0.13 mol) in DMF (10 mL). The acetate groups protecting the carbohydrate hydroxy groups in the glycopeptides were removed manually using 5% hydrazine in DMF (v/v, 5 mL) for 6–15 h. The peptides were cleaved from the resin by treatment with TFA in methylene chloride (2%, v/v, 10 × 5 mL × 5 min) and were collected in a flask containing isopropanol. The peptides were precipitated with cold diethyl ether and the crude products were separated from the remaining solvent by centrifugation. Subsequently, the aglycons were treated with TFA/H_2_O/TIS (95:2.5:2.5) for 30 min after cleavage from the resin. Finally, the peptides were purified by preparative RP-HPLC.

H-[L-Ala-L-Ala-*allo*-L-Thr(GalNAc)]_4_-L-Ala-L-Ala-OH (**5**, 6 mg, 3%): calcd for C_78_H_132_N_18_O_39_, 1945.98 (av.); found, 1944.889917 (monoisotopic); MS (ESI) *m*/*z*: [M + Na]^+^ 1969.2; HRMS (ESI): [M + H + Na]^2+^ 984.44622; [M + 2Na]^2+^ 995.43429.

H-[L-Ala-L-Ala-*allo*-L-Thr]_4_-L-Ala-L-Ala-OH (**6**, 2 mg, 2%): calcd for C_46_H_80_N_14_O_19_, 1133.21 (av.); found, 1132.572420 (monoisotopic); MS (MALDI-TOF) *m*/*z*: [M + Na]^+^ 1155.7; HRMS (ESI): [M + H]^+^ 1133.58309.

H-[D-Ala-D-Ala-D-Thr(GalNAc)]_4_-D-Ala-D-Ala-OH (**7**, 10 mg, 5%): calcd for C_78_H_132_N_18_O_39_, 1945.98 (av.); found, 1944.889917 (monoisotopic); MS (ESI) *m*/*z*: [M + Na]^+^ 1968.0; [M + K]^+^ 1983.9; HRMS (ESI): [M + 2Na]^2+^ 995.43709.

H-[D-Ala-D-Ala-D-Thr]_4_-D-Ala-D-Ala-OH (**8**, 2 mg, 2%): calcd for C_46_H_80_N_14_O_19_, 1133.21 (av.); found, 1132.572420 (monoisotopic); MS (ESI) *m*/*z*: [M + Na]^+^ 1155.4; HRMS (ESI): [M + 2Na]^2+^ 578.28376.

### Microphysical ice recrystallization analysis

The antifreeze activity was determined according to a method described previously. The inhibitory effect of antifreeze agents on ice recrystallization is quantified: The peptides (TFA salts) were dissolved in 45 wt % sucrose solutions in varying concentrations and 2 µL of each of these solutions were placed between glass cover slides (film thickness ~ 10–20 µm). The samples were positioned on a temperature-controlled silver block inside a cold stage (Linkam MDBCS 196) mounted onto an optical microscope (Olympus BX 51) in bright field transmission mode. The samples were cooled down to −50 °C at a rate of 20 °C min^−1^, reheated to −8 °C at a rate of 10 °C min^−1^, and annealed at this temperature for 2 h. The recrystallization of the resulting polycrystalline ice was recorded by taking images with a digital video camera in 12 s time intervals while the images were analyzed simultaneously by using a LabVIEW virtual instrument. The ice recrystallization rate was calculated to obtain the inhibitor related concentration *c*_i_ at which the recrystallization-limiting process turns over from water diffusion to liquid-to-ice transfer due to the presence of ice-binding antifreeze agent [37–38].
